# Supplementary material for: Plasma nanoDSF Denaturation Profile at Baseline Is Predictive of Glioblastoma EGFR Status
Source: Cancers (Basel). 2023 Jan 26;15(3):760. doi: 10.3390/cancers15030760 (PMC9913157; doi:10.3390/cancers15030760)

Supplementary data

Data pre-processing

Raw data from the nanoDSF instrument (F330, F350, A350, F330/F350 ratio, and their respective derivatives) were interpolated using InterpolatedUnivariateSpline from the scipy.interpolate python module in order to ensure the same temperature alignment for all data.

Machine Learning

The Machine Learning code developed for this work uses the scikit-learn toolbox with : (1) LogisticRegression from the linear_model module with parameter max_iter sets to 1000; (2) SVC from the svm module with the following combination of parameters: kernel = “poly”, gamma = “auto”, C = 1, degree = 3; (3) RandomForestClassifier from the ensemble module with parameter n_estimators fixed to 500; (4) AdaBoostClassifier from the module ensemble with the default parameter values except n_estimators = 100. The code and the data used for EGFR amplification detection is publicly available at **https://github.com/RemiEyraud/EGFR_amplification_detection**

Additional Plots

Figure S1: Means of nanoDSF output.

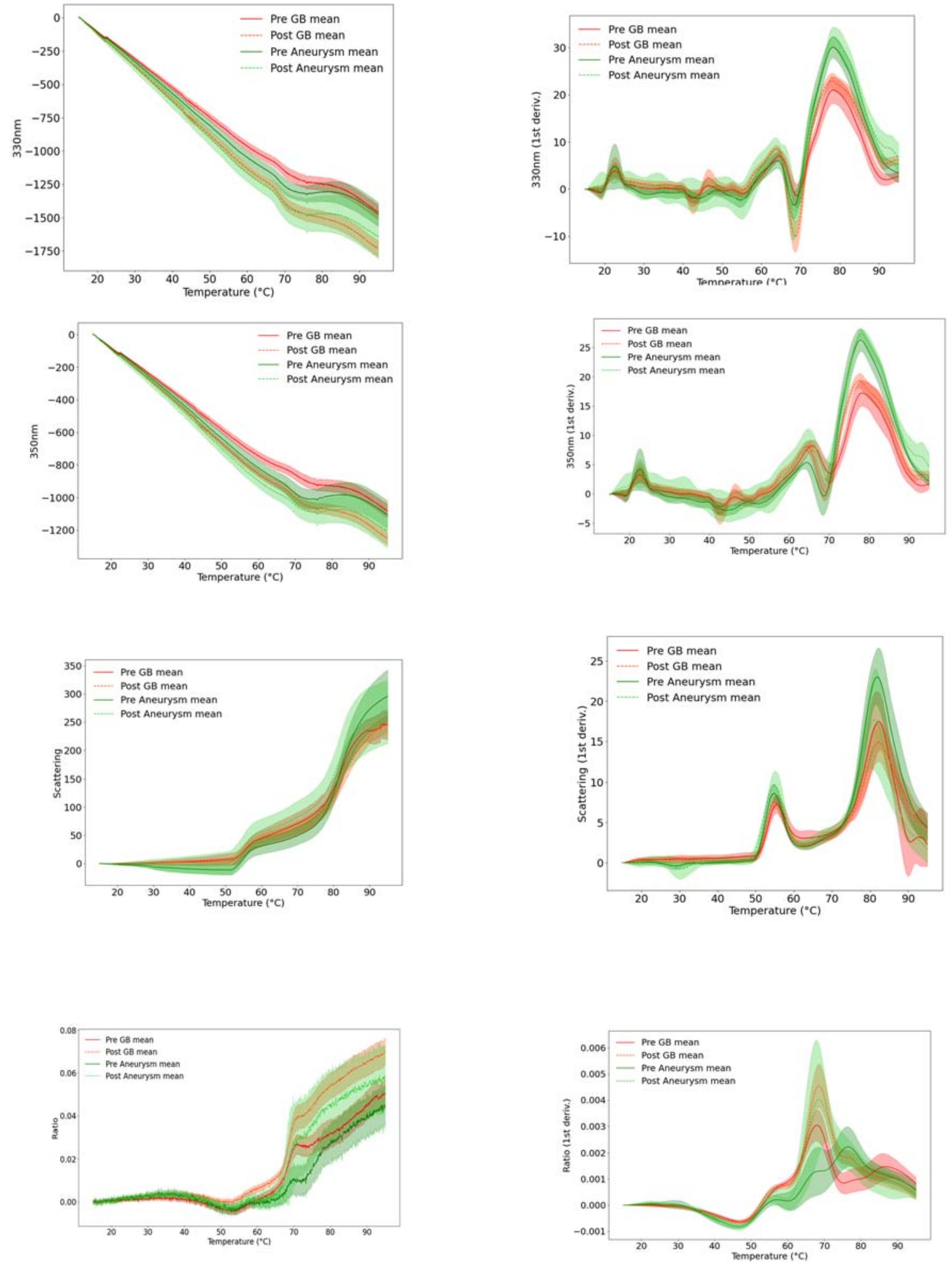

Supplement: Supplementary file 1 [file cancers-15-00760-s001.zip › cancers-1989082-supplementary.pdf]
